# Specific contribution of neurons from the Dbx1 lineage to the piriform cortex

**DOI:** 10.1038/s41598-021-86512-8

**Published:** 2021-04-16

**Authors:** Thando Shabangu, Hung-Lun Chen, Zi-hui Zhuang, Alessandra Pierani, Chien-Fu F. Chen, Shen-Ju Chou

**Affiliations:** 1 Molecular Cell Biology, Taiwan International Graduate Program, Academia Sinica, Taipei, Taiwan; 2 Graduate Institute of Life Sciences, National Defense Medical Center, Taipei, Taiwan; 3 Institute of Cellular and Organismic Biology, Academia Sinica, Taipei, Taiwan; 4 Imagine Institute, Team Genetics and Development of the Cerebral Cortex, Université de Paris, F-75015 Paris, France; 5 Institute of Psychiatry and Neuroscience of Paris, INSERM U1266, Université de Paris, F-75014 Paris, France

**Keywords:** Genetics, Differentiation, Developmental biology, Neurogenesis, Developmental neurogenesis, Neuroscience, Development of the nervous system, Olfactory system

## Abstract

The piriform cortex (PC) is a major cortical processing center for the sense of smell that receives direct inputs from the olfactory bulb. In mice, the PC consists of three neuronal layers, which are populated by cells with distinct developmental origins. One origin of PC neurons is the pool of Dbx1-expressing neural progenitors located in the ventral pallium at the pallial-subpallial boundary. Since the precise mechanisms of PC neuron development are largely unknown, we sought to define the distribution, timing of neurogenesis, morphology and projection patterns of PC neurons from the Dbx1 lineage. We found that Dbx1-lineage neurons are preferentially distributed in layer 2 and enriched in the ventral portion of the PC. Further, Dbx1 neurons are early-born neurons and contribute to most neuronal subtypes in the PC. Our data also revealed an enrichment of Dbx1-lineage neurons in the ventral anterior PC that project to the orbitofrontal cortex. These findings suggest a specific association between the developmental origin of PC neurons and their neuronal properties.

## Introduction

Odors are potent signals that can convey information through time and space^1^. To process these signals, mammals generally rely on an olfactory system comprised of a three-level neural pathway that includes a sensor sheet (the olfactory epithelium; OE), a primary processing level (main olfactory bulb; MOB), and a secondary processing level (the olfactory cortex). The connections between sensory neurons of the OE and principal neurons of the MOB follow wiring principles that depend on sensory neuron receptor choice. Thus, a stereotypic afferent organization is found in the MOB^2^, which can explain topographic odor representation at the primary processing level. Beyond the MOB, odor information is dispatched via axons of the principal neurons, mitral and tufted cells, to several paleo- and neocortical areas, which are together called the olfactory cortex (OC). The projections of mitral and tufted cells in individual glomeruli do not show apparent spatial preferences in the OC^3,4^, leading to highly distributed odor representation in most OC areas^5–8^. While the overall organization of the olfactory system is known, how the OC contributes to odor perception is still an open question.

The piriform cortex (PC) is the largest area in the OC, and it receives a complete set of MOB projections^3^. The PC is divided into two anatomically and functionally distinct sub-regions, the anterior PC (APC) and the posterior PC (PPC)^9,10^. Additionally, based on cytoarchitecture and connectivity, the APC can be further divided into dorsal and ventral subdomains. These distinct PC subregions have different input and output projection patterns ^10–12^. For example, the ventral APC receives inputs from both mitral and tufted cells in the olfactory bulb, while dorsal APC and PPC only receive inputs from the mitral cells^9,13,14^. Furthermore, the projection neurons in the ventral APC predominantly project to the lateral orbital cortex^9,15^. Based on these specific inputs and outputs, the PC sub-regions likely contribute differently to olfactory processing.

Cortical columns in the PC consist of three neuronal layers. The outermost layer is called layer 1 (L1); it is a superficial plexiform layer, containing axons of mitral and tufted cells mostly within L1a and association fibers from other PC and cortical areas in L1b^16^. Layer 2 (L2) is the most cell-dense layer in the PC, and it is further subdivided into an outer layer 2a (L2a) and inner layer 2b (L2b). L2a is predominantly occupied by semilunar cells, while L2b consists mainly of pyramidal neurons. Compared to L2, layer 3 (L3) has a relatively low density of neurons, most of which are large pyramidal neurons. In contrast to the six-layer neocortex, the developmental process of the three-layer piriform cortex remains poorly understood, at least partially due to the fact that PC neurons originate from multiple sources, including both the lateral and ventral pallium^17–19^.

In this study, we sought to define the contribution of a specific lineage, the Dbx1 (developing brain homeobox 1) lineage, in the PC. Dbx1 is a homeodomain transcription factor involved in neuronal fate specification^20–22^. During early corticogenesis, Dbx1 is highly expressed in the preoptic area (POA), in the septum and ventral pallium (VP) at the pallial-subpallial border (PSB) in the forebrain; notably, its expression in the VP is greatly reduced after E14.5^23^. Previous studies showed that neurons of the Dbx1-lineage contribute to PC^19,23,24^. Here, we first used a genetic model with an enlarged PC to demonstrate that the Dbx1 expression level is correlated with the size of PC. This finding suggests that neurons of the Dbx1 lineage are important contributors to the PC. Using *Dbx1*^*Cre*^ to label neurons from the Dbx1 lineage, we confirmed that this population contributes significantly to the PC. We further characterized the distribution, morphology, and neurogenesis patterns of the PC neurons derived from the Dbx1 lineage and found these neurons show stereotypical distributions, timings of neurogenesis and output projection patterns. Our findings suggest that neuronal lineage might be a critical determinant of PC functional domains.

## Results

### The expression of Dbx1 is correlated with the size of PC

**Figure 1:**
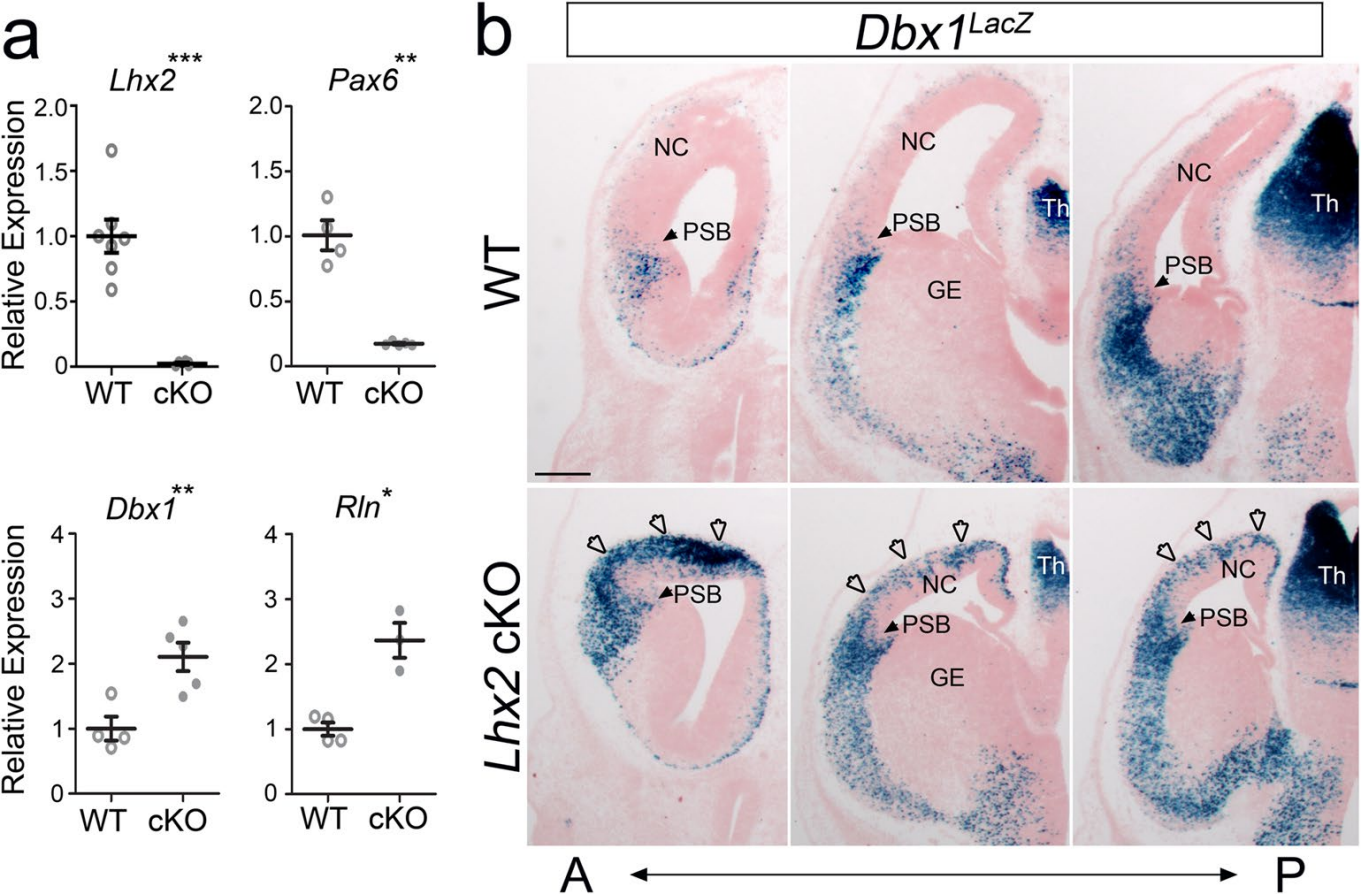
Dbx1 is upregulated in Lhx2 conditional knockout cortices. (**a**) According to qPCR, the expression levels of *Lhx2* and *Pax6* are significantly downregulated in the cKO (*Lhx2*^f/f^; *Emx1*^*Cre*^) cortex when compared with WT (*Lhx2*^*fl/*+^) at E13.5, while the levels of *Dbx1* and *Rln* are significantly upregulated in cKO mice (n = 3–5; *Lhx2*, *P* < 0.001; *Pax6*, *P* = 0.0054; *Dbx1*, *P* = 0.0085; *Rln*, *P* = 0.0412). (**b**) LacZ staining on coronal sections of *Dbx1*^*LacZ*^ cortices of WT (top) and cKO (bottom) embryos at E13.5. From anterior (A) to posterior (P), LacZ^+^ cells (blue) are specifically located in the pallial–subpallial boundary (PSB, arrowheads) in WT. An increased number of LacZ^+^ cells (indicated by unfilled arrowheads) was observed in the neocortex (NC) of the cKO. Scale bar, 200 µm. GE, ganglionic eminence; Th, thalamus.

A dramatic expansion of the Dbx1 expression domain was previously reported in the dorsal telencephalon of *Lhx2* null mutant cortices^25^. As neurons derived from the *Dbx1* lineage contribute to the PC^19^, and deletion of *Lhx2* in cortical progenitors by *Emx1*-Cre leads to the generation of ectopic piriform cortex^26^, we further tested whether the expanded PC in *Lhx2* conditional knockout animals (*Lhx2* cKO; *Lhx2f.*^/f^:*Emx1*^*Cre*^) is correlated with an increase of *Dbx1* expression. We first confirmed that the expression of *Lhx2* is absent and the expression of *Pax6* is down-regulated in the dorsal telencephalon of cKO cortices at E13.5, as shown previously^27^. We then compared the expression levels of *Dbx1* in control and *Lhx2* cKO cortices. Using qPCR, we found that *Dbx1* and *Reelin* (*Rln*), a marker for Cajal Retzius cells (which arise from the *Dbx1* lineage^28^), are both significantly up-regulated in the *Lhx2* cKO cortex (Fig. 1a).

To confirm that the *Dbx1* expression pattern is expanded in *Lhx2* cKO mice, we crossed the *Lhx2* cKO with a *Dbx1*^*LacZ*^ reporter line, which has a *LacZ* gene knocked-in at the *Dbx1* locus^20^. While LacZ-expressing cells are derived from the VP at PSB in wild-type *Dbx1*^*LacZ*^ animals, we observed that the number of *Dbx1*-LacZ-expressing cells was dramatically increased in the *Lhx2* cKO at E13.5 (Fig. 1b). Further, we found many LacZ^+^ cells were distributed throughout the cKO dorsal telencephalon (Fig. 1b), in accordance with the dorsal expansion of the PC in *Lhx2* cKO mice. This correlation between increased production of *Dbx1*-lineage neurons and increased PC size in the *Lhx2* cKO suggests that the *Dbx1* lineage could contribute significantly to the PC.

### Dbx1-lineage neurons in the piriform cortex are mostly excitatory neurons

**Figure 2:**
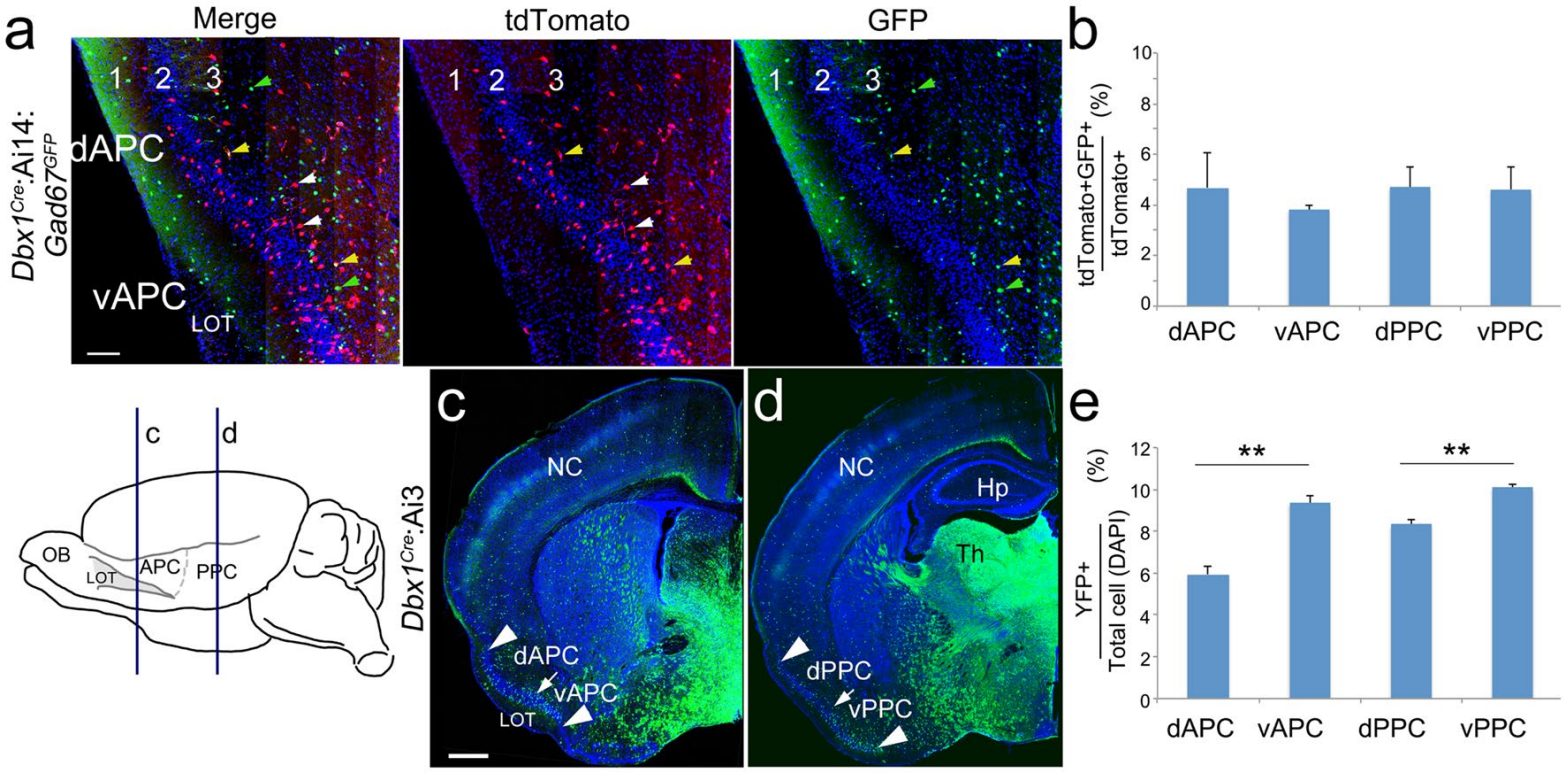
The contribution of cells from the *Dbx1* lineage in piriform cortex. (**a**) Coronal sections at the level of the APC from *Dbx1*^*Cre*^:Ai14:*Gad67*^*GFP*^ cortex at P30. Cells of the *Dbx1* lineage were labeled by tdTomato, and inhibitory interneurons were labeled by GFP. GFP-positive and tdTomato-positive cells are indicated by green and white arrowheads respectively, while double-labeled cells are indicated by yellow arrowheads. (**b**) Quantification of the percentage of interneurons in the Dbx1 lineage (number of GFP^+^tdTomato^+^ cells/number of tdTomato^+^ cells). About 4% of the *Dbx1* lineage cells are inhibitory interneurons, across the PC (n = 3). (**c**, **d**) Coronal sections of *Dbx1*^*Cre*^:Ai3 cortex at P7 at the level of the anterior piriform cortex (APC) and posterior PC (PPC), as indicated on the left. Cells of the *Dbx1* lineage were labeled by YFP (green); nuclei were stained with DAPI (blue). The APC and PPC are further divided into dorsal and ventral subregions. Arrowheads mark the dorsal and ventral ends of the PC, and arrow marks the border between dorsal and ventral PC. (**e**) Density of cells of the *Dbx1* lineage (number of YFP + cells/number of total cells) measured in a radial column in PC subregions. *Dbx1* cell denisty is significantly higher in ventral PC regions (n = 3). LOT, lateral olfactory tract; OB, olfactory bulb; NC, neocortex; Hp, hippocampus. Scale bars, 100 µm (**a**) and 500 µm (**c**).

Next, we performed a basic characterization of the Dbx1-lineage neurons in the PC. In addition to its expression in the VP, Dbx1 is also expressed in the POA, and the *Dbx1* lineage contributes to both excitatory and inhibitory neurons in the amygdala^29^. Thus, we first determined what portion of the *Dbx1*-derived cells in the PC are GABAergic interneurons. To identify inhibitory interneurons among the *Dbx1*-derived cells in the PC, we crossed *Dbx1*^*Cre*^:Ai14 mice, in which *Dbx1*-lineage cells are labeled with tdTomato, with the *Gad67*^*GFP*^ transgenic line, where all GABAergic inhibitory interneurons are labeled with GFP^30^. At P30, we quantified the *Dbx1*-derived cells (tdTomato^+^) and *Dbx1*-derived interneurons (tdTomato^+^GFP^+^), and we calculated the percentage of *Dbx1*-lineage neurons that are interneurons (Fig. 2a). We found a relatively low percentage (~ 4%) of *Dbx1*-derived cells to be GFP^+^ across the PC (Fig. 2b), suggesting that very few *Dbx1*-derived cells in the PC are GABAergic inhibitory neurons.

We also examined the proportion of *Dbx1*-derived cells in the *Emx1*-lineage. Emx1 is expressed in cortical progenitors located within the ventricular zone of the dorsal telencephalon, and most excitatory projection neurons in the cerebral cortex are derived from this lineage^31^. As we could not label distinct lineages with two Cre lines in one animal, we used the *Dbx1*^*LacZ*^ reporter line to trace cells of *Dbx1* lineage in *Emx1*^*Cre*^: Ai14 cortices, where cells of *Emx1* lineage express tdTomato. At E12.5, we found that along the anterior/posterior axis, the majority of the *Dbx1*-LacZ^+^ cells around the PSB (~ 80%) also express tdTomato (Figure S1). This high degree of colocalization suggests that most cells derived from *Dbx1* progenitors are excitatory neurons of the *Emx1* lineage.

### Preferential ventral distribution of Dbx1-derived neurons in the piriform cortex

We next studied the distribution of Dbx1 neurons by dividing the PC into four sub-regions, including the dorsal and ventral portions of APC and PPC. As can be seen in Fig. 2c, the APC appears as an elongated “S” shape in coronal sections of *Dbx1*^*Cre*^:Ai3 (allows permanent tracing of *Dbx1*-derived cells) cortex at P7. The dorsal and ventral portions of the APC were identified by the presence of the lateral olfactory tract (LOT) above L1 specifically in the ventral APC (vAPC). In contrast with the APC, the PPC does not have a superficial LOT, and its structure is more linear (Fig. 2d). The dorsal and ventral halves of the PPC were denoted as dPPC and vPPC, respectively. After crossing the *Dbx1*^*Cre*^ mice^23^ with the Ai3 reporter line to label *Dbx1*-derived cells with YFP, we measured the *Dbx1*-derived cell density in PC at P7 and found that cells derived from the *Dbx1* lineage contributed to about 8% of all PC cells. Further, when we compared *Dbx1*-derived cell density in dorsal and ventral APC and PPC, we found significantly higher densities of *Dbx1*-lineage cells (*Dbx1*-lineage cells (green)/total cells labeled by DAPI) in the ventral APC and PPC regions, compared with the corresponding dorsal regions (dAPC, 5.94 ± 0.41%; vAPC, 9.36 ± 0.32%; n = 3; *P* = 0.0027. dPPC, 8.32 ± 0.23%; vPPC, 10.09 ± 0.13%; n = 3; *P* = 0.0025) (Fig. 2e).

**Figure 3:**
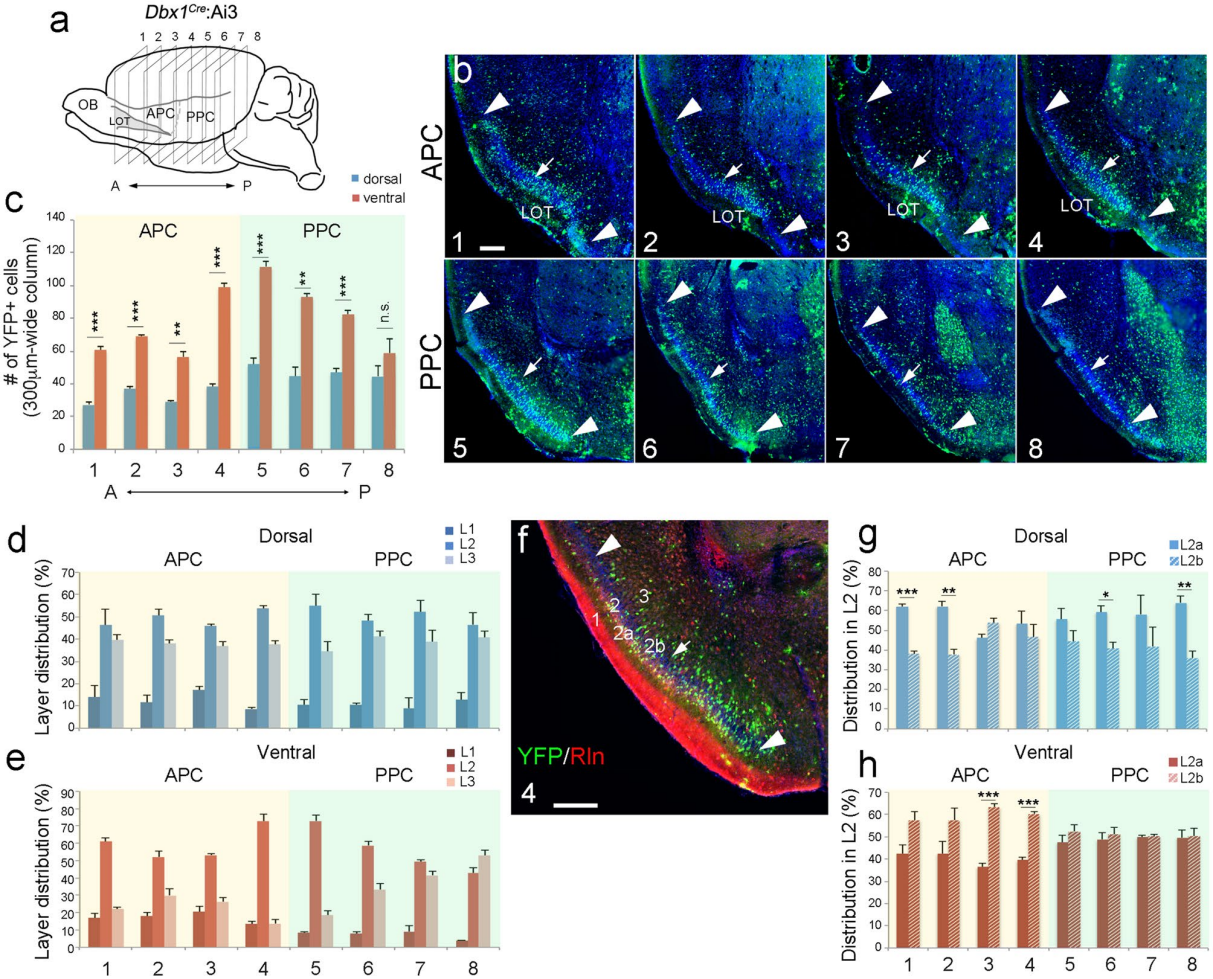
Neurons of the *Dbx1* lineage show spatial and laminar distribution preferences in PC. (**a**, **b**) Coronal sections of *Dbx1*^*Cre*^:Ai3 cortex at P7 at eight levels from anterior (A) to posterior (P). Arrowheads mark the dorsal and ventral ends of PC and arrow marks the border between dorsal and ventral PC. (**c**) Quantification of the number of YFP-expressing cells in dorsal and ventral PC at each level. Significantly higher numbers of YFP^+^ cells were found in the ventral PC compared to dorsal PC (level 1, *p* = 0.00036; level 2, *p* = 1.0085E-05; level 3, *p* = 0.00165; level 4, *p* = 2.442E-05; level 5, *p* = 0.00042; level 6, *p* = 0.00101; level 7, *p* = 0.00059; level 8, *p* = 0.2561; n = 3). (**d**, **e**) Quantification of laminar distribution of YFP^+^ cells. Most YFP^+^ cells were detected in layer 2 (L2), across the PC. (**f**) Immunostaining for Reelin (red) on coronal section of *Dbx1*^*Cre*^:Ai3 cortex (at level 4 as in c). Reelin staining is enriched in L2a. (**g**, **h**) Within L2, the percentages of YFP^+^ cells in L2a and L2b are shown across the PC (n = 3). Scale bar, 100 µm.

To further determine how cells of the *Dbx1* lineage are distributed across the PC, we collected serial coronal sections of P7 *Dbx1*^*Cre*^:Ai3 cortices and quantified the numbers of YFP-expressing cells in the PC at eight evenly distributed planes along the anterior–posterior axis (as shown in Fig. 3a). By comparing the total number of *Dbx1*-derived neurons in each section, we found that most *Dbx1*-derived cells are located in the middle of the PC, including the caudal APC and rostral PPC (e.g., sections 4 and 5 in Fig. 3b). Consistent with our observation in Fig. 2e, most sections had significantly more *Dbx1*-derived cells in the ventral PC than in the dorsal regions (Fig. 3b, c); within a 300-µm-wide column, the numbers of *Dbx1* derived cells were roughly twice as high in the ventral PC than in the dorsal PC, for most of the sections across the PC (Fig. 3c). Thus, our data showed that neurons of the *Dbx1* lineage are preferentially distributed toward the ventral PC.

Furthermore, we analyzed the laminar distribution of *Dbx1*-derived cells by quantifying the percentage of *Dbx1*-derived cells in each layer across the PC. Although *Dbx1*-derived cells were found in all layers, the majority were found in L2 and L3, with more than 50% of the *Dbx1*-derived neurons located in L2 in most sections (Fig. 3d, e). In the dorsal APC, PPC and posterior ventral PPC, *Dbx1*-derived neurons were similarly distributed in L2 and L3 (Fig. 3d, e). However, in the ventral APC, the *Dbx1*-derived neurons were highly enriched in L2 (Fig. 3e).

L2 can be further divided into L2a and L2b. We thus examined the distribution of *Dbx1*-derived cells in L2a and L2b, using Reelin expression to demarcate L2a^32–34^ (Fig. 3f). At many levels in the dorsal PC, more *Dbx1*-derived cells were detected in L2a than in L2b (Fig. 3g). However, in the ventral APC, more *Dbx1*-derived cells were found in L2b, and in the ventral PPC, similar numbers of *Dbx1*-derived cells were found in L2a and L2b (Fig. 3h).

### Dbx1-lineage neurons show diverse neuronal morphologies

**Figure 4:**
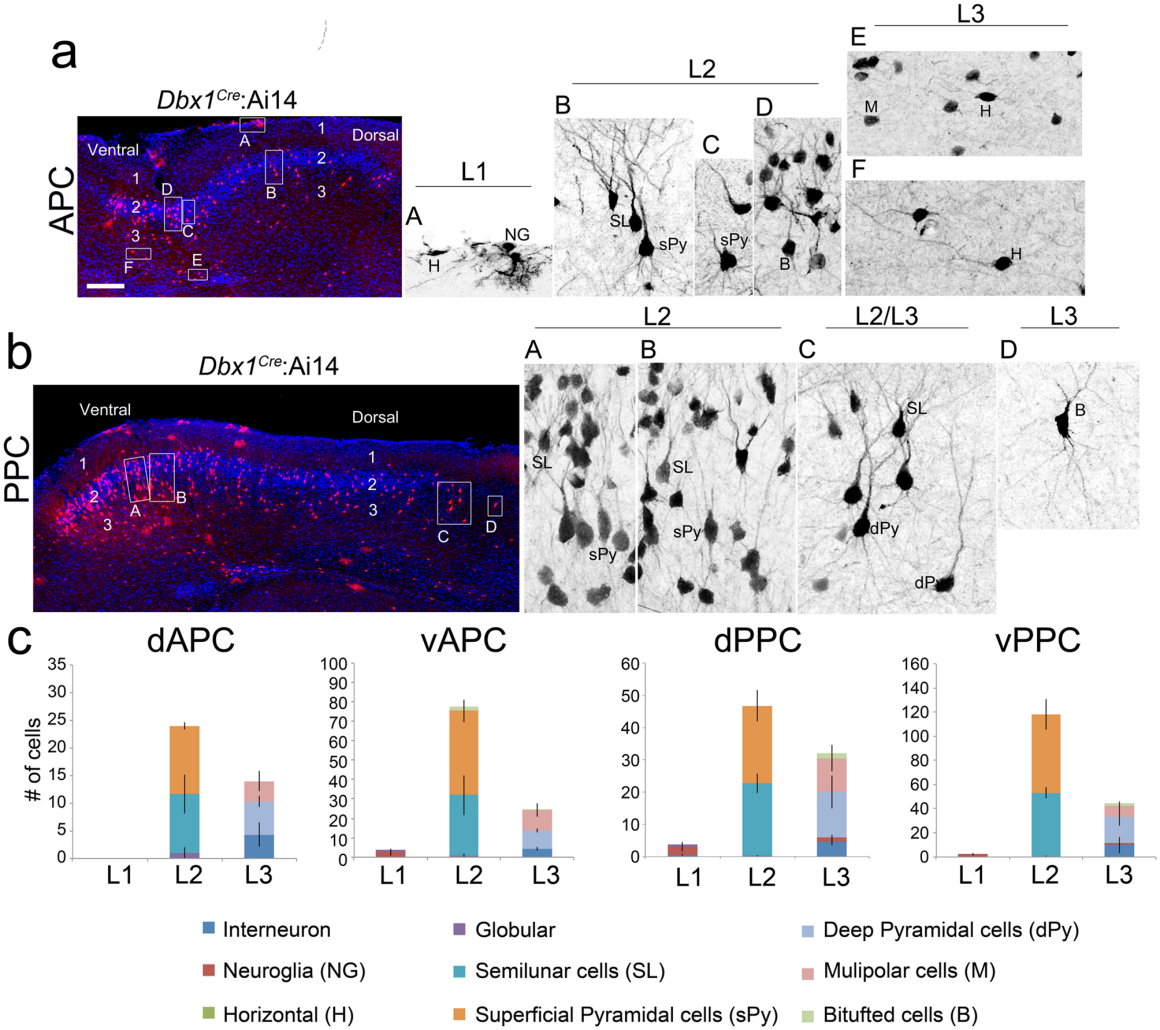
Morphologies of neurons of the *Dbx1* lineage. (**a**, **b**) Morphologies of neurons from the *Dbx1* lineage were observed in coronal sections of the APC (**a**) and PPC (**b**) of *Dbx1*^*Cre*^:Ai14 cortices at P30. Higher magnifications of insets are shown on the right. Different cell types were identified based on their morphologies. (**c**) Quantification is shown for cells of each type in different sub-regions of the APC and PPC (n = 3). Scale bar, 100 µm.

After determining the distributions, we sought to assess the cell types of the *Dbx1*-derived cells, according to their morphology (as described in the Methods). In the PC, several neuronal subtypes have been described based morphology, including horizontal cells in L1, semilunar cells and superficial pyramidal cells in L2, and deep pyramidal cells and multipolar cells in L3^35^. To characterize the morphologies of *Dbx1*-lineage cells in the PC, we crossed *Dbx1*^*Cre*^ with Ai14 and took advantage of the strong tdTomato expression in *Dbx1*-derived cells at P30. Based on cellular morphology, we saw very few *Dbx1*-derived cells were glia and the majority were neurons at the analyzed stages. As shown in Fig. 4, the *Dbx1* lineage exhibits a variety of neuronal morphologies throughout the PC. Similar to the cell types reported in previous studies, we observed *Dbx1* cells with morphologies corresponding to horizontal cells (H) and neuroglia (NG) in L1 (Fig. 4a), semilunar cells (S) and superficial pyramidal cells (SPy) in L2 (Fig. 4a, b), and deep pyramidal cells (DPy) and multipolar cells (M) in L3 (Fig. 4a, b).

We then quantified the numbers of *Dbx1*-derived neurons belonging to each neuronal subtype. Although the *Dbx1*-derived cell densities differed among different PC subregions, the composition of different cell types was relatively similar throughout the PC. In L1, *Dbx1*-derived cells were mostly neuroglia and horizontal cells. In L2, about half the *Dbx1*-derived cells were semilunar cells, and the other half were superficial pyramidal cells. In L3, the *Dbx1* neurons were predominantly deep pyramidal cells and multipolar cells (Fig. 4c).

### Early generation of Dbx1-lineage cells

We further sought to delineate the developmental stages at which the *Dbx1* cells are generated. Therefore, we injected EdU into pregnant *Dbx1*^*Cre*^:Ai3 mice at specific developmental time-points from E11.5 to E14.5 to label neurons generated at each stage. We first investigated the pattern neurogenesis in the general population of PC neurons. Then, we compared this general pattern to that of *Dbx1*-derived PC neurons.

**Figure 5:**
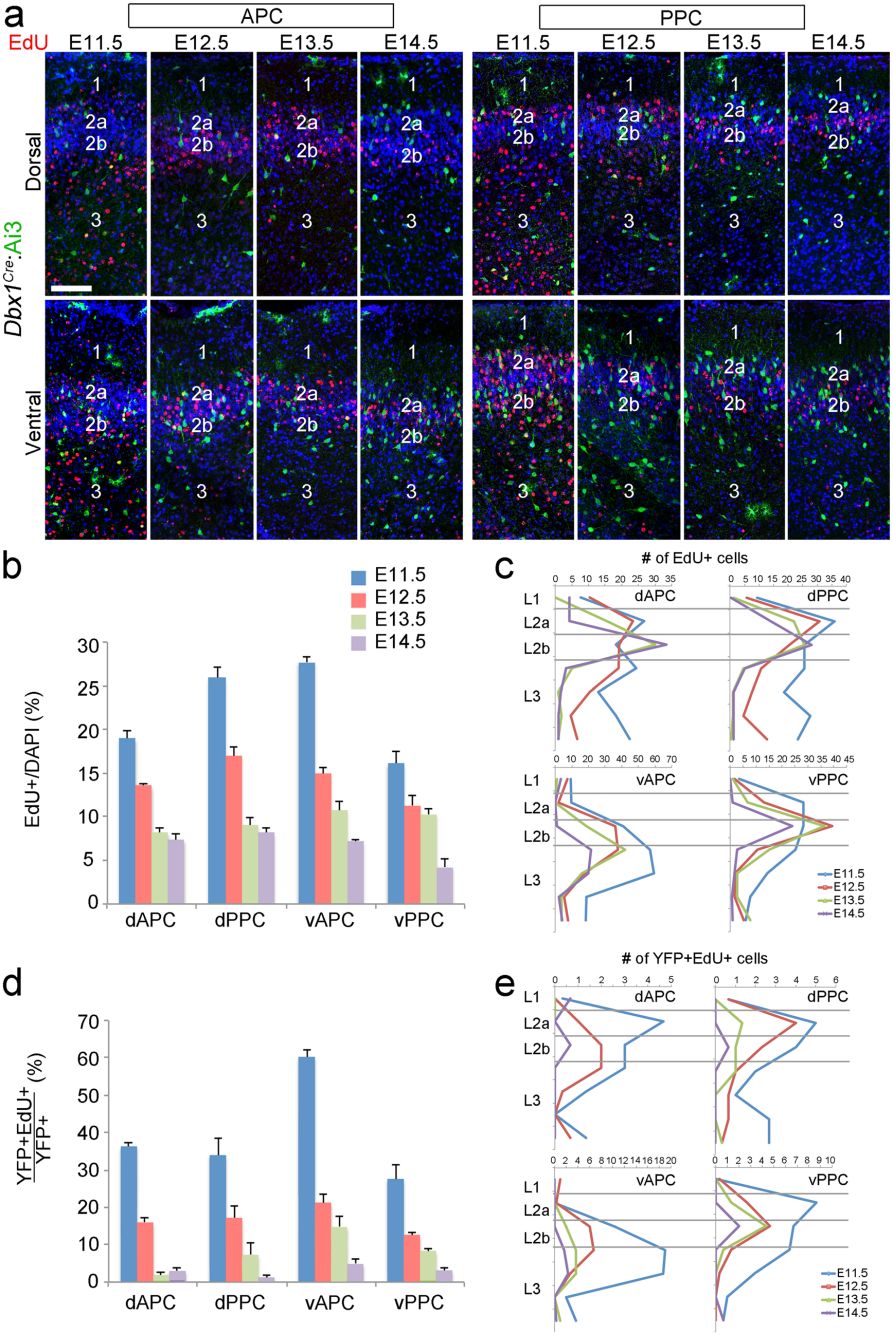
Neurons of the *Dbx1* lineage are early born neurons in PC (**a**) YFP (cells of the *Dbx1* lineage, green) and EdU staining (red) in coronal sections from P7 *Dbx1*^*Cre*^:Ai3 mice. EdU was injected into pregnant mothers at indicated stages. (**b**–**e**) Quantification of results from (**a**), indicating the percentages of EdU^+^/total cells (indicated by DAPI) (**b**) and EdU^+^YFP^+^/total YFP^+^ cells (**d**). The numbers and distributions of EdU^+^ cells and EdU^+^YFP^+^ cells at the indicated stages were shown in (**c**) and (**e**), respectively (n = 3). Scale bar, 100 µm.

We analyzed the number and distribution of EdU^+^ cells in the dorsal and ventral APC and PPC at P7. In agreement with previous studies^33,36–38^, we found that neurogenesis was initiated by E11.5 in the PC (Fig. 5a, b). Comparing the samples from different time-points, we found the number of cells produced decreases over time in all compartments of the PC. For example, significantly more cells were generated at E11.5 than at E12.5-E14.5 (Fig. 5b), and the number of cells produced after E14.5 was greatly diminished. We also compared the laminar distribution of cells generated at each developmental stage. In contrast to the regular inside-out neurogenesis pattern observed in the neocortex^39^ (Figure S2), the pattern of neurogenesis was more complicated in the PC. Cells produced at E11.5 were distributed throughout L2 and L3 in PC, but most cells generated from E12.5 to E14.5 contributed to L2 (Fig. 5a, c), demonstrating an overall inside-out neurogenesis pattern. However, within L2, earlier born cells were generally located in L2a, which is more superficial than later born cells (Fig. 5c) and represents an outside-in pattern. Thus, our results agreed with previous reports of dual inside-out/outside-in neurogenesis gradients in the developing PC^33,36–38,40^. Further, we found the laminar distribution patterns of cells generated between E11.5 and E14.5 were similar among different domains in the PC (Fig. 5c).

We then analyzed the number and distribution of the *Dbx1*-lineage cells (YFP^+^) generated between E11.5 and E14.5 at P7. Compared to the general cell population in the PC, most *Dbx1*-derived cells were generated at earlier time-points. The number of *Dbx1* cells produced also decreased over time in all compartments of the PC. When we compared the neurogenesis patterns of *Dbx1*-derived cells to the entire population of cells in the PC, we found a significantly higher percentage of *Dbx1* cells generated at E11.5 in the APC (Fig. 5d, e). For example, in the dorsal APC, about 20% of all cells are generated at E11.5, but a significantly higher percentage of *Dbx1* cells (about 35%) are generated at E11.5 (*P* = 0.000261, n = 3). Similarly, 27% of cells in the ventral APC are generated at E11.5, while a significantly higher percentage of Dbx1 cells (about 60%) in this region are generated at E11.5 (*P* = 0.000115, n = 3) (Fig. 5d). Additionally, a much lower percentage of *Dbx1* cells across the PC are generated after E14.5, when compared with PC cells in general (Fig. 5d, e). We found the distributions of *Dbx1* cells generated at specific stages were similar to PC cells in general; cells generated at E11.5 were distributed in both L2 and L3, while cells generated at and after E12.5 were more focused in L2 (Fig. 5a, e). Notably, we observed that the majority of Dbx1 cells generated at E11 in vAPC accumulated in deep L2 instead of superficial L2, as was the case in dAPC and both PPC areas.

Overall, the neuronal birthdating analyses demonstrated that cells of the *Dbx1* lineage generated between E11.5 and E14.5 show a similar distribution pattern to the general PC neuronal population. However, the *Dbx1* derived PC cells contribute mainly to the early-born population of PC cells; most were generated by E11.5 or E12.5, and almost no *Dbx1*-derived cells were generated after E14.5.

### Many Dbx1-derived neurons in the vAPC project to the orbitofrontal cortex

**Figure 6:**
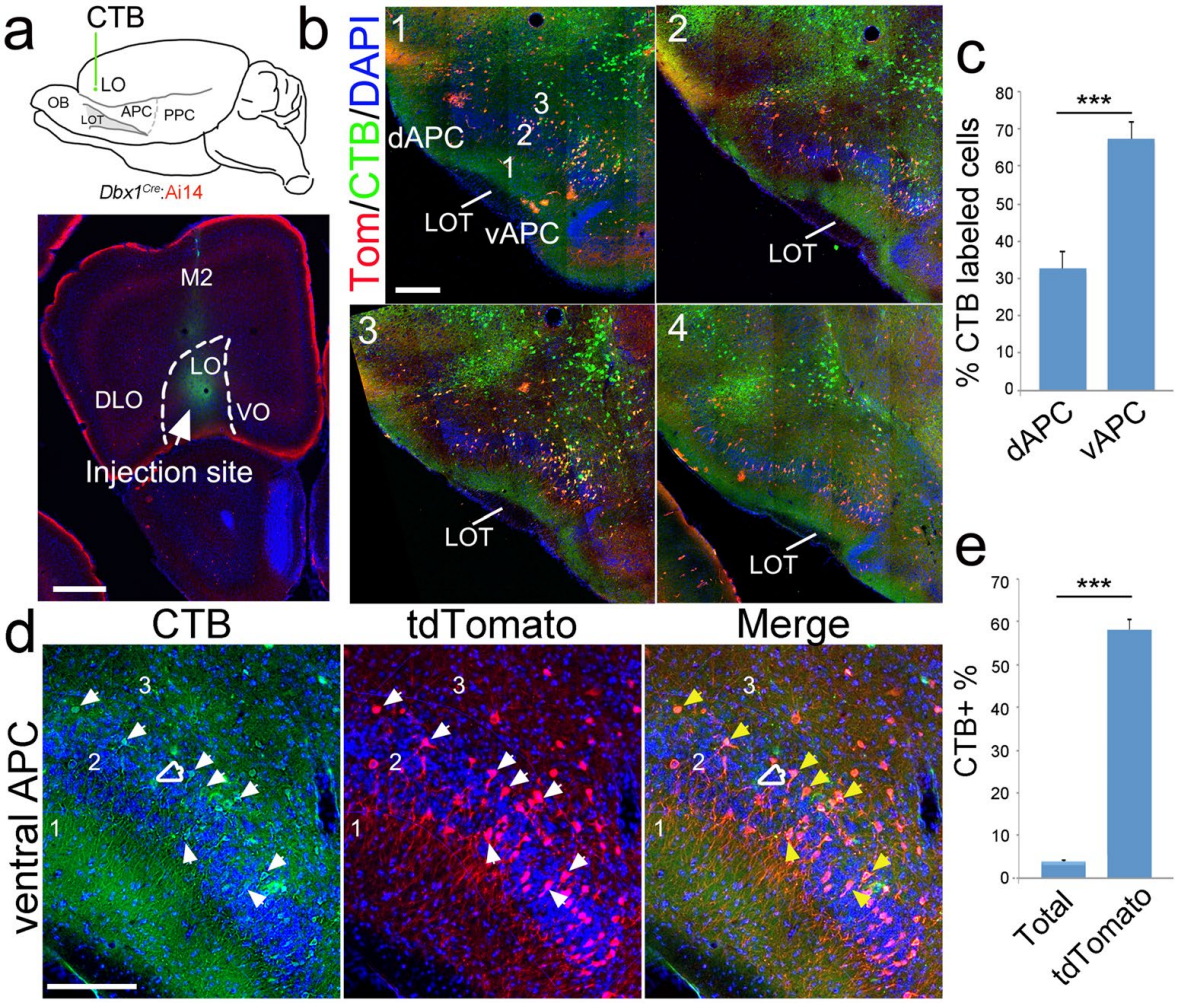
Neurons of the *Dbx1* lineage in the ventral APC preferentially project to the orbitofrontal cortex. (**a**) Green fluorescence (Alexa Fluor 488)-conjugated CTB was injected into the lateral orbitofrontal cortex (LO) in *Dbx1*^*Cre*^:Ai14 mice at P30. An injection site is shown in the coronal section (below). (**b**) CTB-labeled neurons (green) were detected throughout the APC (sections 1–4 from anterior to posterior). Neurons of the *Dbx1* lineage were labeled by tdTomato (Tom + , red). (**c**) Significantly more CTB-labeled neurons were detected in the ventral APC than in the dorsal APC. (**d**) LO-projecting neurons (green) were enriched in L2 of the ventral APC. Green-only neurons are indicated by hollow arrowheads and yellow neurons (positive for both green and red) are indicated by solid arrowheads. (**e**) Among the total population of neurons in vAPC L2 (labeled by DAPI), about 4% were LO projection neurons (labeled by CTB). Significantly higher percentage of LO-projecting neurons was found within the neuronal population of the *Dbx1* lineage (more than 50%) (n = 3). Scale bar, 500 µm (**a**), 100 µm (**b**) and 50 µm (**d**).

Next, we examined the efferent projections of the *Dbx1*-lineage neurons. Since neurons of the *Dbx1* lineage were preferentially distributed in the ventral PC (Fig. 3) and orbitofrontal cortex-projecting neurons are also preferentially located in the ventral PC^15^, we investigated whether *Dbx1* neurons preferentially project to the orbitofrontal cortex. A retrograde neural tracer, cholera toxin B subunit (CTB) coupled to green fluorescent dye, was injected into the lateral orbitofrontal cortex (LO) of *Dbx1*^*Cre*^:Ai14 mice (Fig. 6a). In agreement with previous findings^15^, we found many LO-projecting neurons (CTB^+^) in the APC, with a majority located in the vAPC (Fig. 6b, c). We further quantified the number of CTB positive and CTB and tdTomato double positive neurons in the section containing the most CTB^+^ neurons in the ventral APC. Interestingly, the majority of LO-projecting neurons in the vAPC were derived from the *Dbx1* lineage (CTB^+^tdTomato^+^) (Fig. 6d) (CTB^+^tdTomato^+^/number of CTB^+^ cells: 72.98 ± 7.22%; n = 3). Among the general population of neurons in the vAPC, only about 4% were CTB^+^ (number of CTB^+^ cells/number of total cells, labeled by DAPI: 3.91 ± 0.20%) (Fig. 6e). However, a significantly higher proportion of neurons of the *Dbx1* neuronal lineage (tdTomato +) were CTB + (number of CTB^+^tdTomato^+^/number of tdTomato^+^ cells: 58.13 ± 2.29%; *P* < 0.001, n = 3, when compared to the general population of neurons in the vAPC) (Fig. 6e). This finding suggested that neurons of *Dbx1* lineage in the vAPC preferentially project to the orbitofrontal cortex.

## Discussion

The PC has long been considered synonymous with the primary olfactory cortex^41^, as it is critical for processing of external chemical signal inputs to influence perception, emotion, learning and memory. The PC is also an important hub for delivering odor information to higher centers, such as the prefrontal cortex, hippocampus and amygdaloid areas^42^. In this study, we analyzed the contribution of *Dbx1*-lineage neurons to the PC. In the spinal cord, differential regional expression of *Dbx1* along the ventral-dorsal axis is known to be critical for the differentiation of V0 and V1 neuronal fate^20^. *Dbx1* is also expressed in a specific progenitor population in the VP at the PSB^23^. We showed that very few *Dbx1*-lineage cells in the PC are interneurons or glia. Although *Dbx1*-derived cells are found in all layers of the PC across the anterior–posterior axis, and the neurons exhibit layer-specific idiosyncratic neuronal morphologies, we identified a distinct generation time-point for *Dbx1*-lineage cells, which corresponds to their most frequent laminar fate in the PC. We found *Dbx1*-lineage cells were born relatively early among the PC neuronal population. As neuronal birthdates were shown to determine neuronal physiology and connectivity in the dentate gyrus^43^, the birthdating results for *Dbx1*-derived neurons suggest that these cells might have specialized roles in the PC. However, whether neuronal birthdates determine cellular and/or functions in PC requires additional study.

A specific function for *Dbx1*-lineage neurons was further suggested by their distribution preferences, i.e., we found the *Dbx1*-derived cell number peaks at the middle of PC, and the cells are enriched in L2 and the ventral PC. Further, the *Dbx1*-lineage neurons in the ventral APC show a significant preference for projection to the lateral orbitofrontal cortex. Thus, our findings that neurons derived from the *Dbx1* lineage show a preferred distribution, timing of neurogenesis and output projection pattern, suggest that these cells might contribute to a specific PC neuronal population or a specific function of the PC.

The differential distribution of *Dbx1*-lineage neurons in different PC subregions could partially contribute to the different functions in these PC subregions. The demarcation of the PC into subregions with functional differences was hypothesized decades ago^44,45^. However, this topic has been minimally explored. The APC was initially divided into dorsal and ventral regions based on structural differences, such as the presence of the LOT in the vAPC. Additionally, specific stimulation of these two regions elicited different physiological outcomes^9,44,45^. In particular, a deep region in the ventral APC was found to be a sensitive area for stimulation-evoked seizures^46^. Our findings that neurons in vAPC are generally produced earlier than those in dAPC and that *Dbx1*-lineage cells in vAPC are born early among the overall vAPC cell population suggest that the timing of neurogenesis might also contribute to the functional demarcation between PC subregions.

A pertinent open question is how the PC processes olfaction. In the olfactory bulb, each mitral and tufted cell sends a single dendrite to its respective glomerulus, and it sends axon collaterals to multiple higher brain areas, including the PC^4,47^. Individual PC neurons receive projections from mitral and tufted cells throughout the OB, making odor representations in the PC not only spatially distributed but also spatially intermingled^5,48^. With afferent circuits highly distributed, a recent study revealed that the PC efferent circuit appears to be topographically organized^15^. Chen and colleagues showed that PC neurons with different orbitofrontal targets have distinct and stereotypic distribution patterns across the PC, and these two neuronal populations overlap minimally. This finding suggests that neurons with the same output targets might serve as functional units of the PC. However, the mechanisms that determine these output projection patterns of PC neurons were previously unknown. Our findings suggest that neuronal lineage/origin might be involved determining efferent projection patterns for PC neurons. This idea is consistent with a previous hypothesis that the connectivity of PC neurons is specified by neuronal molecular identity^32^.

Taken together, the preference of *Dbx1*-lineage neurons for ventral distribution, early neurogenesis and projection output to the lateral orbitofrontal cortex suggests that neuronal lineage could partly contribute to determining the functional properties of PC neurons. Thus, our study provides a better understanding of PC organization, and it could ultimately contribute to the understanding of the neural mechanisms underlying odor percept formation.

## Methods

### Mouse lines

*Dbx1*^*LacZ*^^18^ and *Dbx1*^*Cre*^^23^ mouse lines were kindly provided by Dr. Alessandra Pierani. Lhx2 floxed, *Emx1*^*Cre*^ and *Gad67*^*GFP*^ mice were kindly provided by Drs. Dennis O’Leary^26^, Kevin Jones^31^, and Yuchio Yanagawa^30^. Additional reporter lines, such as Ai3 [Gt(ROSA)26Sor^tm3(CAG-EYFP)Hze^] and Ai14 [Gt(ROSA)26Sor^tm14(CAG-tdTomato)Hze^]^49^, were used. The day of identifying a vaginal plug and the day of birth were respectively designated as embryonic day 0.5 (E0.5), and postnatal day 0 (P0). Animal care and experimental procedures were approved by and performed in accordance with guidelines provided by the Academia Sinica Institutional Animal Care and Use Committee. The reporting in this manuscript follows the recommendations in the ARRIVE guidelines.

### Quantitative RT-PCR

Quantitative RT-PCR was performed as described^50^. Briefly, RNA samples were collected from dorsal telencephalon at E13.5 using TriPure Isolation Reagent (Roche). After treated with DNaseI (Promega), 1 μg total RNA was subjected for first-strand cDNA synthesis with Transcriptor First Strand cDNA Synthesis Kit (Roche) and 0.2 μl cDNA was used for each quantitative PCR reaction. Real-time RT-PCR was performed using LightCycler 480 SYBR Green I Master mix (Roche). Gene expression was normalized to GAPDH and data were analyzed by two-tailed, unpaired *t*-test with Welch’s correction.

Primers used for qPCR: GAPDH, F: GGCAAATTCAACGGCACAG, R: CGGAGATGATGACCCTTTGG; Lhx2, F: GCATCTACTGCAAAGAAGACTACTACA, R: CGCATCACCATCTCT GAGG; Pax6, F: GCCCTTCCATCTTTGCTTGGGAAA, R: TAGCCAGGTTGGGA AGAACTCTG; Reelin, F: AACCACGGCCTTACATGG, R: GTAAATTCCTGGCA GCTTGG; Dbx1, F: CAACAGACCCACCACCTTCT, R: AGGAGCTGGCACTCTG AAA.

### Immunohistochemistry and EdU labeling

Timed-pregnant mice were dissected, and embryonic cortices were fixed in 4% phosphate-buffered paraformaldehyde (PFA); postnatal brains were perfused with and postfixed in 4% PFA. For histological analyses, brains were cryoprotected with 30% sucrose in PBS, embedded in Tissue-Tek OCT compound (Sakura Finetek) and cut in 20–25 μm sections on a cryostat (Leica). Immunohistochemistry was performed as described^26^. In short, primary antibodies, including chick anti-mCherry (Abcam, ab205402, 1:500) and anti-GFP (Torey Pines Biolabs, TP-401, 1:500) were incubated overnight at 4 °C in blocking solution containing 3% BSA (Sigma-Aldrich) and 0.3% Triton X-100 in phosphate buffer, followed by incubation with Alexa-conjugated secondary antibodies (Jackson ImmunoResearch) for 2 h at room temperature. Cell nuclei were counterstained with DAPI (Vector). Neuronal birthdating analyses were performed as described^50^. Briefly, EdU (500 ng) was injected into timed-pregnant mice, and the EdU-positive cells were detected with a Click-iT EdU imaging kit (Life Technologies).

### CTB tracing

Animals (young male *Dbx1*^*Cre*^:Ai14 mice on a C57BL/6 J background; ~ 30 g, 4–5 weeks old) were anesthetized by injecting ketamine/xylazine (initial dose, 90 mg/10 mg/kg) intraperitoneally. A deeply anesthetized animal was placed into a stereotaxic device with a heating unit. One burr hole (∼3 mm × 2 mm) was drilled on the dorsal surface of the skull. Dura mater was carefully removed using a new 19 G needle. The injection micropipette was pulled from glasses capillaries (OD, 1.14 mm; ID, 0.53 mm, Drummond Scientific Company) with an opening of ∼20 μm in diameter. The micropipette installed onto a pressure injector (Nanoject III, Drummond Scientific Company) was loaded with 0.5% CTB, Alexa Fluor 488 conjugate (ThermoFisher Scientific) in phosphate buffer saline (pH 7.4). The micropipette tip was then positioned into the LO (2.46 mm anterior and 1.25 mm lateral to bregma and 1.50 mm from the surface) to deliver 50 nl of CTB solution at the speed of 1 nl/s. The mice were fully anaesthetized and perfused with 4% (wt/vol) paraformaldehyde 7 days after CTB injection. The collected brains were kept in 30% sucrose solution and then cryosectioned coronally at 35 µm.

### Quantification of cellular morphology

The morphological characterization of neuronal subtypes in PC was performed using coronal sections of P30 *Dbx1*^*Cre*^, Ai14, *Gad67*^*GFP*^ cortices at the levels of APC and PPC. The morphologies of all tdTomato^+^ cells in PC were analyzed, according to previously established criteria^51,52^. In short, in L1, horizontal cells have cell bodies oriented parallel to the pial surface, with dendritic trees that are perpendicular to the apical and basal spines of pyramidal cells; neuroglial cells have a glia-like appearance, with small dendritic branches; pyramidal cells have a pyramidal shape, with an apical axon projecting towards the pial surface and a basal tree of spiny dendrites directed towards the deeper layers of the cortex. Superficial pyramidal cells are located in L2, and deep pyramidal cells are located in L3. The size and length of the apically projecting axon of a superficial pyramidal cell is smaller than that of a deep pyramidal cell. The semilunar cells, located in L2a, lack the basal dendritic tree projecting downwards. Globular cells are globular in appearance and have dendritic spines radiating around the globular sphere. Multipolar cells in L3 do not have a clear axonal projections but have dendritic arborizations extending in multiple directions and restricted to L3. Bitufted cells have an apical and basal orientation with two prominent axons, one projecting towards the pial surface and one directed away. Interneurons were distinguished by their expression of GAD67-GFP^30^.

### Quantification and statistical analyses

In general, 300-µm-wide cortical columns were cropped for quantification of cell numbers and marker intensity. The numbers of YFP^+^, tdTomato^+^, and EdU^+^ cells were manually counted using ImageJ FIJI. All analyses were performed with three or more biological replicates. The number of individual animals of the same genotype used is indicated as “n” in the text and figures. Statistical analyses were performed using GraphPad Prism 5 software. All quantitative data are presented as the mean ± SEM. Minimal statistical significance was fixed at *P* < 0.05 for comparisons made by unpaired t-test with Welch’s correction. Significance is represented in figures as: *P < 0.05; **P < 0.01; ***P < 0.001.

## Supplementary Information

Supplementary Information
