# Supplementary material for: Specific contribution of neurons from the Dbx1 lineage to the piriform cortex
Source: Sci Rep. 2021 Apr 16;11:8349. doi: 10.1038/s41598-021-86512-8 (PMC8052341; doi:10.1038/s41598-021-86512-8)
Supplement: Supplementary file 1 — Supplementary Information [file 41598_2021_86512_MOESM1_ESM.pdf]

Supplementary information for:

Specific contribution of neurons from the Dbx1 lineage to the piriform cortex

Thando Shabangu[1, 2, 3], Hung-Lun Chen[2], Zi-hui Zhuang[1], Alessandra Pierani[4,5], Chien-Fu F. Chen[2,3,*], and Shen-Ju Chou[1,3,*]

1. Institute of Cellular and Organismic Biology, Academia Sinica, Taipei, Taiwan
2. Graduate Institute of Life Sciences, National Defense Medical Center, Taipei, Taiwan
3. Molecular Cell Biology, Taiwan International Graduate Program, Academia Sinica, Taipei, Taiwan
4. Université de Paris, *Imagine* Institute, Team Genetics and Development of the Cerebral Cortex, F-75015, Paris, France
5. Université de Paris, Institute of Psychiatry and Neuroscience of Paris, INSERM U1266, F-75014, Paris, France

*Corresponding authors:

Shen-Ju Chou

Institute of Cellular and Organismic Biology, Academia Sinica,

128 Academia Rd. Sec. 2, Taipei, 11529, Taiwan.

Tel: 886-2-2789-9530

Email: schou@gate.sinica.edu.tw

Chien-Fu F. Chen

Graduate Institute of Life Sciences, National Defense Medical Center,

No.161, Sec. 6, Minquan E. Rd., Taipei,114, Taiwan

Tel: 886-2-8792-3100 ext:18572

Email: t70cyy@yahoo.com

Figure S1

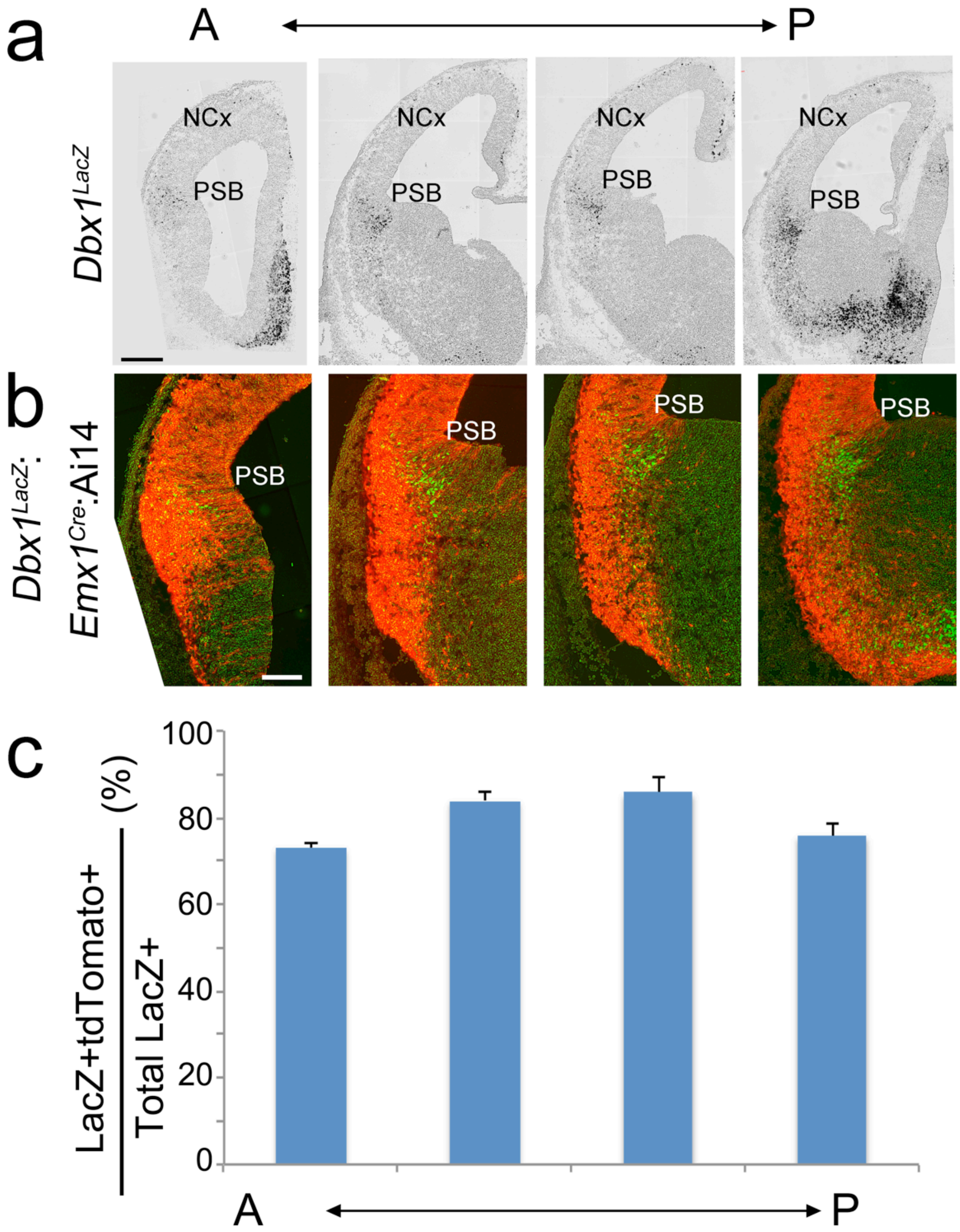

Figure S1. The majority of *Dbx1* lineage neurons are projection neurons of the *Emx1* lineage. (a) LacZ staining on coronal sections of *Dbx1*^{LacZ} cortices at E12.5. LacZ+ cells are found in pallial-subpallial boundary (PSB). (b) High level of overlap was found in the expression of b-Gal (green) and tdTomato (red) in *Dbx1*^{LacZ}: *Emx1*^{Cre}: Ai14 cortices at E12.5. (c) Quantification of the percentage of tdTomato expressing cells in cells expressing *Dbx1*-LacZ on sections from anterior (A) to posterior (P). More than 70% of the *Dbx1*-LacZ expressing cells are derived from the Emx1 lineage. Scale bars, 200μm.

Figure S2

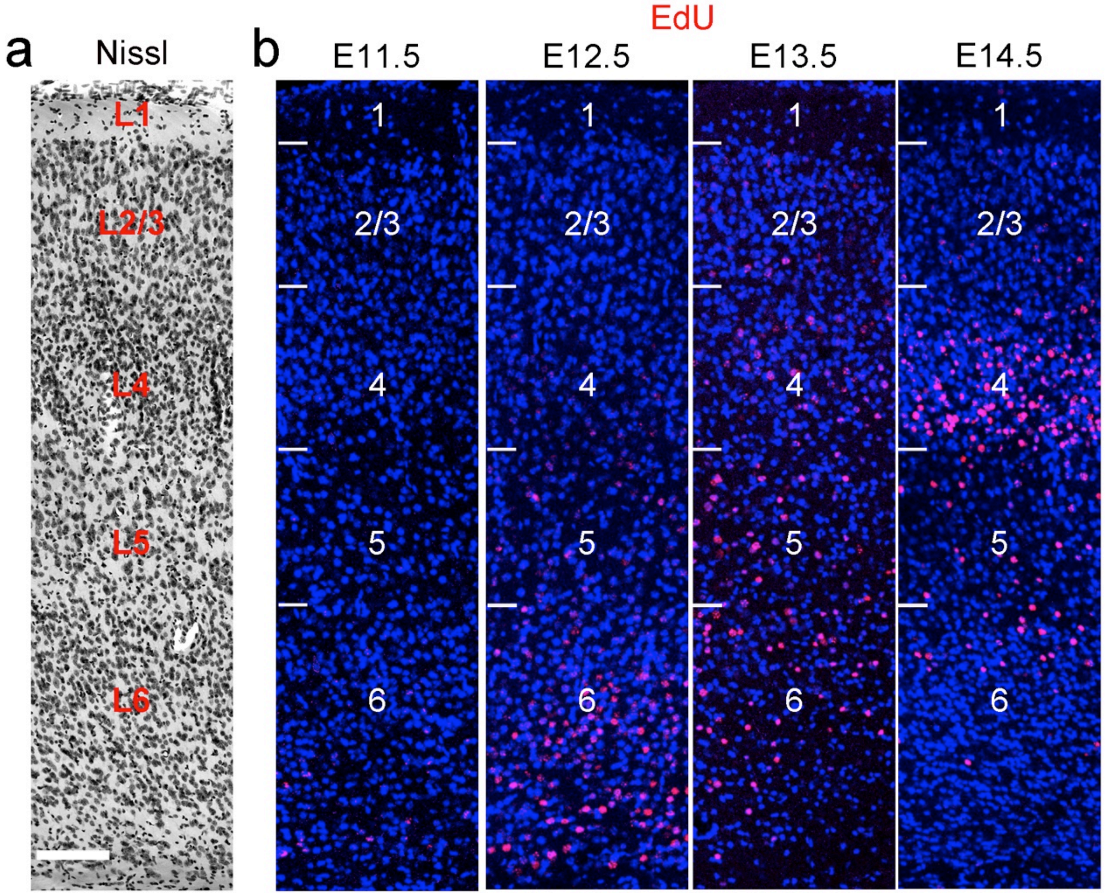

Figure S2. Inside-out neurogenesis in the neocortex. (a) Six cortical layers in the neocortex are visualized by Nissl staining on a coronal section of a wild type cortex at P7. (b) EdU administered from E11.5 to E14.5 labeling neurons generated that these stages. EdU positive cells (red) showed an inside-out neurogenesis pattern in the neocortex at P7: earlier born neurons are in deeper layers than later born neurons. Scale bars, 100μm.
